# General and Symptom‐Specific Illness Duration and Course as Predictors of Symptom Severity in Anorexia Nervosa

**DOI:** 10.1002/eat.24525

**Published:** 2025-10-02

**Authors:** Kira G. Venables, Ariana R. Bazzi, Abigail Smith, Soo‐Eun Lee, Carol B. Peterson, Ann F. Haynos

**Affiliations:** ^1^ Department of Psychology Virginia Commonwealth University Richmond Virginia USA; ^2^ Center for Deployment Psychology Uniformed Services University of the Health Sciences Bethesda Maryland USA; ^3^ Department of Medical and Clinical Psychology Uniformed Services University of the Health Sciences Bethesda Maryland USA; ^4^ Henry M. Jackson Foundation for the Advancement of Military Medicine, Inc. Bethesda Maryland USA; ^5^ Department of Clinical Mental Health Counseling Richmont Graduate University Chattanooga Tennessee USA; ^6^ Department of Psychiatry and Behavioral Sciences University of Minnesota Minneapolis Minnesota USA

**Keywords:** anorexia nervosa, assessment, eating disorders, illness course, illness duration

## Abstract

**Objective:**

Illness duration has been examined as a predictor of anorexia nervosa (AN) outcomes to mixed results, yet is frequently used to make treatment and prognosis decisions. More specific metrics, such as prior illness course (e.g., continuous vs. intermittent symptoms) and symptom‐specific duration or course, may more effectively predict concurrent and longitudinal outcomes.

**Method:**

Adults with acute or weight‐restored AN (*N* = 75) completed a measure assessing duration and course (e.g., continuous, intermittent) of their eating disorder in general and of specific symptoms (low weight, restrictive eating, weight/shape preoccupation). Hierarchical linear regressions predicted the severity of each symptom (BMI, restrictive eating, shape concern, weight concern) at baseline and one‐year follow‐up from covariates (Step 1), general illness duration and course (Step 2), and symptom‐specific duration and course (Step 3).

**Results:**

Cross‐sectionally, symptom‐specific predictors improved model fit for all outcomes (*p*s = < 0.001–0.043). A more continuous course of restrictive eating was associated with more frequent baseline restrictive eating (*p* = 0.002) and longer duration (*p* = 0.009) and a more continuous course (*p* < 0.001) of weight/shape preoccupation was associated with higher baseline shape concern. General illness duration and course did not predict symptom severity after accounting for symptom‐specific factors. Longitudinally, no measure of illness or symptom duration or course predicted follow‐up symptom severity.

**Conclusions:**

Courses of specific symptoms may be more meaningful symptom severity markers in AN than general illness duration or course, particularly for restriction and shape concern. Findings highlight the clinical utility of assessing symptom‐specific chronicity and of interrupting eating disorder symptoms in treatment, given the impact of continuous symptom expression.

**Summary**

- We investigated the ability of different measures of illness chronicity to predict clinical symptoms among adults with anorexia nervosa (AN).
- Continuity of specific symptoms (e.g., restriction, shape concern) may be better markers of current severity than general illness duration; further, no measure of illness chronicity predicted long‐term outcomes.
- Findings may enhance assessment of AN severity, encourage symptom interruption, and provide hope that improvement is possible for those with chronic symptoms.

## 1 Introduction

Anorexia nervosa (AN) is a severe eating disorder associated with numerous and sometimes life‐threatening psychological and physiological consequences (Puckett et al. 2021). Courses of illness in AN are highly variable: while some individuals fully recover following the onset of symptoms, others fluctuate between periods of relapse and remission, and still others experience chronic symptom trajectories without significant recovery episodes (Romano et al. 2020; Bardone‐Cone et al. 2018). Relapse is common, with an estimated 40%–50% of patients who undergo intensive treatment for AN returning to intensive care within 10 years (Carter et al. 2012; Khalsa et al. 2017), yet predictors of eating disorder severity or prognosis remain unclear (Sala et al. 2023).

Illness duration, which is defined as the amount of time elapsed since an individual first developed an eating disorder, independent of the pattern of symptom expression over time, is one variable that has been frequently examined as a predictor of AN symptom severity and long‐term outcomes to mixed results. Some studies have found that longer illness duration in AN is associated with greater symptom severity and relapse risk (Fichter et al. 2006, 2017; Keel and Brown 2010; Meule et al. 2023). However, others have failed to support associations between illness duration and AN symptom severity, relapse rates, or treatment response (Ranzenhofer et al. 2022; Raykos et al. 2018). Notably, two recent meta‐analyses found that eating disorder duration was not significantly associated with relapse risk (Sala et al. 2023) or treatment response (Radunz et al. 2020), indicating that the empirical validity of this construct requires further examination. Further, although enduring AN can be debilitating, longitudinal studies suggest that most individuals with AN eventually recover, even those with protracted illness trajectories (Eddy et al. 2017; Eielsen et al. 2022). Thus, illness duration alone may not be an effective predictor of clinical severity and symptom outcomes in AN.

Despite mixed evidence regarding the predictive validity of illness duration, this construct is frequently used to make critical decisions regarding illness prognosis, treatment, and mechanisms (Davis et al. 2020; Meule et al. 2023; Takakura et al. 2019; Webb et al. 2022). There is a long‐standing assumption that longer illness durations are associated with worse prognosis in AN, which can impact treatment decisions. For instance, the term “severe and enduring” AN has been used to describe individuals with more protracted and severe illness (Broomfield et al. 2017; Touyz and Hay 2015). It has been hypothesized that individuals who meet criteria for severe and enduring AN due to longer illness duration may need alternate treatment approaches (Ålgars et al. 2023; Marcolini et al. 2024) or may be unlikely to experience meaningful symptom improvement and, therefore, may need palliative care (Wonderlich et al. 2020). At the most extreme, the diagnostic specifier of “terminal AN” has been proposed. In one case series, this specifier was used to make recommendations about medical aid in dying to individuals with severe and enduring AN older than 30 years who, despite having participated in eating disorder care, endorsed the belief that additional treatment was futile and that death from symptom complications was inevitable (Gaudiani et al. 2022). Thus, illness duration is frequently employed to make even the most crucial decisions regarding the conceptualization and treatment of AN, despite mixed evidence regarding its concurrent or predictive validity. For this reason, it is of vital importance that the operationalization and measurement of this construct be evaluated.

Inconsistency or inadequacy in defining and measuring illness duration may contribute to these mixed findings. Studies often do not report how illness duration was measured, reflecting the lack of standardized measures of these constructs. In studies that do report this information, illness duration is often operationalized using single‐question measures (e.g., “How long have you had an eating disorder?”) that rely on participants' interpretation of what constitutes the start of their eating disorder (Fernández‐Aranda et al. 2021; Sala et al. 2023). Additionally, questions about illness duration alone may not adequately assess symptom continuity (i.e., whether an individual has experienced ongoing or interrupted symptoms). As a result, an individual with a certain illness duration (e.g., 20 years) may have experienced continuous symptoms throughout the entirety of that period, whereas another individual may have experienced a short‐lived illness when younger and recently relapsed after a long period of remission. It is likely that illness duration will hold different informative value in these two cases.

Measures that broadly assess overall illness duration also do not capture information about the lifetime duration and course of specific *symptoms* (e.g., restrictive eating, unhealthy low weight, or weight and shape preoccupation). Subthreshold disordered eating commonly precedes full‐threshold eating disorder onset (Ranzenhofer et al. 2022); additionally, different eating disorder symptoms may emerge and remit throughout one's life (Neumark‐Sztainer et al. 2010). Thus, more specific measures of the duration and course of individual eating disorder symptoms in AN may provide a more granular perspective than illness duration alone. For instance, singular symptoms, such as body mass index (BMI) at discharge from intensive treatment, have been found to predict AN relapse likelihood over illness duration (Redgrave et al. 2021). Examining AN course from this “zoomed‐in” approach has the potential to highlight symptom patterns that may be useful in more accurately assessing severity and relapse risk.

Thus, we aimed to further investigate the utility of using prior illness characteristics to inform current severity and predict future outcomes, while improving on limitations of the prior literature. In this study, we examined the ability for general AN illness duration and course (i.e., continuity versus discontinuity of symptoms), as well as symptom‐specific (i.e., restrictive eating, unhealthy low weight, and weight/shape preoccupation) duration and course, to concurrently and longitudinally predict symptom severity in an adult sample. We tested the following pre‐registered hypotheses: (1) Symptom‐specific illness duration and course variables would be cross‐sectionally associated with the severity and frequency of the specific associated eating disorder symptom (e.g., restrictive eating duration and course would be associated with restrictive eating frequency) above the effects of general eating disorder duration and course, and would exhibit stronger relations with the severity of the associated current symptom than general illness course or duration; and (2) Symptom‐specific duration and course variables would longitudinally predict the severity and frequency of the associated eating disorder symptom above the effects of general eating disorder duration and course, and would exhibit stronger relations to the severity of the associated future symptom than general illness course or duration.

## 2 Methods

### 2.1 Participants

The current study is a secondary analysis of data collected at the University of Minnesota for five neuroimaging or treatment studies investigating reward and emotion‐based mechanisms of AN. These included four cross‐sectional studies and one 12‐month longitudinal study with baseline, 3‐, 6‐, 9‐, and 12‐month follow‐up visits (only 12‐month is examined in this analysis). Individuals from the Minneapolis/St. Paul area were recruited through print and digital advertisements in treatment centers, the community, and social media. Participants were adults (*N* = 75) with acute AN (*n* = 31) or weight‐restored AN (AN‐WR; *n* = 44) diagnosed using the Structured Clinical Interview for DSM‐5 Research Version (SCID‐5‐RV; First et al. 2015) or the Mini International Neuropsychiatric Interview (MINI; Sheehan et al. 1998). The sample included participants with both restricting (*n* = 62) and binge eating/purging (*n* = 13) AN subtypes. Due to participant overlap among the five parent studies, any participants who completed more than one study (*n* = 21) were only included once in the dataset, from the first of the studies in which they participated. Supplement A details the parent studies and their inclusion/exclusion criteria.

### 2.2 Procedure

At baseline, all AN and AN‐WR participants completed measures assessing the duration and course of their eating disorder and specific eating disorder symptoms, as well as current eating disorder severity. Height and weight were objectively measured either by the study team or an outside provider. Longitudinal follow‐up was conducted on a subset of AN‐WR participants (*n* = 32) who responded to the same clinical measures at a follow‐up visit 12 months after baseline. The remainder of the participants did not have 12‐month follow‐up data available due to the design of the parent study procedures. All procedures for the original studies were approved by the University of Minnesota Institutional Review Board, and all participants provided written informed consent before engaging in study procedures. Current study hypotheses and analysis plans were pre‐registered at https://osf.io/5ncuw.

### 2.3 Measures

#### 2.3.1 Demographics

At baseline, participants self‐reported demographic characteristics including age, gender, race/ethnicity, and current treatment enrollment. Age and current treatment were selected as covariates in our analyses, as younger age and treatment engagement have been associated with more favorable outcomes in AN (Solmi et al. 2021). Race/ethnicity, gender, and education were reported descriptively, but not included as covariates due to less established links to eating disorder outcomes and limited variability on these metrics within the sample.

#### 2.3.2 Duration and Course of Illness

The Course of Illness Scale, adapted from the Structured Interview for Anorexic and Bulimic Disorders (SIAB; Fichter et al. 1998), is a self‐report questionnaire assessing general and symptom‐specific eating disorder duration and course. Participants reported the age at which they believed their eating disorder began and the age at which they first experienced each specific symptom (e.g., restrictive eating, unhealthy low weight, weight/shape preoccupation). Illness and symptom‐specific durations were measured by subtracting participants' current age from the age at which they reported first having an eating disorder or the specific symptom. The original SIAB has been shown to be reliable and valid in samples with acute and weight‐restored AN (Fichter et al. 1998; Fichter and Quadflieg 2001).

This measure also queries about the course or pattern of illness and each individual symptom. Course of illness and each specific symptom were measured on a Likert‐type scale ranging from 0 to 4 and accompanied by visual representations of the illness course (Supplement B). Response options were: 0 = *“No [symptom]”*; 1 = “Short episodes of [symptom] followed by longer intervals without [symptom]. Overall, [symptom] has been present 30% of the time or less”; 2 = “Periods of [symptom] and periods without of approximate length. Overall, [symptom] has been present about 30%–60% of the time”; 3 = “Long periods of [symptom] with only short intervals without [symptom]. Overall, [symptom] has been present 60%–90% of the time”; and 4 = “Chronic [symptom]. [Symptom] has been present about 90%–100% of the time. There have been no major intervals without [symptom].” In our analyses, this measure was treated continuously; higher scores indicated more continuous symptom course. However, Supporting Information Table 1 provides descriptive information on categorical responses.

For participants who did not endorse a specific symptom, the duration and course of that symptom were set at 0. In this study, we examined the duration and course of participants' eating disorder (general illness duration) and of the specific symptoms of restrictive eating, unhealthy low weight, and preoccupation with weight/shape. Other symptoms (e.g., binge eating and purging) captured on this measure were not examined due to the relatively low endorsement of these symptoms across the sample.

#### 2.3.3 Clinical Measures

##### 2.3.3.1 BMI

Objective height and weight were measured at baseline to calculate BMI. Measurement occurred in person when possible on a calibrated scale and stadiometer. For study visits completed remotely (e.g., due to the COVID‐19 pandemic), participants' healthcare provider confirmed objective height and weight. When objective weights were unavailable for follow‐up visits, the participant self‐reported current weight. Prior research has demonstrated that individuals with AN can provide valid self‐reported estimates of their weight (Glasofer et al. 2020).

##### 2.3.3.2 Restrictive Eating

The Dietary Restriction Screener (DRS; Haynos and Fruzzetti 2015) is a brief measure assessing the presence and frequency of restrictive eating behavior over the past month. The DRS defines restrictive eating, provides examples, and asks participants to indicate whether they have engaged in restrictive eating in the past month. As in prior research (Wang et al. 2021), we used a version of the DRS that assessed past‐month restrictive eating frequency. The DRS has been found to predict eating disorder symptoms, intended and actual food intake, and clinical severity in several studies (Haynos and Roberto 2017; Wang et al. 2018), including predicting reduced objective in vivo food intake (Haynos and Fruzzetti 2015). The DRS was used to measure restrictive eating behavior at each assessment.

##### 2.3.3.3 Weight and Shape Concern

The Eating Disorder Examination (EDE; Cooper and Fairburn 1987) assessed eating disorder symptoms at baseline and follow‐up. The EDE is an investigator‐based, semi‐structured interview assessing eating disorder psychopathology that contains the following subscales: Restraint, Eating Concern, Weight Concern, and Shape Concern. The EDE has been shown to have strong reliability and validity (Berg et al. 2012). For the current study, the Weight Concern and Shape Concern subscales of the EDE were used to assess weight concern and shape concern, respectively.

### 2.4 Data Analysis

Analyses were conducted in R. For longitudinal analyses, if participants were missing a key study measure at 12‐month follow‐up, their last measurement on that variable was carried forward from a prior follow‐up visit. Participants who only completed cross‐sectional procedures (*n* = 43) were excluded from longitudinal analyses. Exploratorily, we examined descriptive information for eating disorder and symptom‐specific duration and course and conducted inter‐item correlations to examine the potential relations between variables.

Hierarchical linear regression models evaluated whether the lifetime duration and course of each specific symptom (restrictive eating, unhealthy low weight, and weight/shape preoccupation) predicted severity of the corresponding symptom (restrictive eating frequency, BMI, and weight/shape concern, respectively) at baseline, controlling for general illness duration and course and covariates. These models were repeated to predict follow‐up values on the same outcomes for those who completed 12‐month follow‐up visits. The regression models included the following steps: (1) Covariates (age, current treatment) and baseline values for outcome variables in longitudinal models; (2) General illness duration and course were entered as predictors; and (3) Symptom‐specific duration and course were entered as predictors.

Dependent variables included BMI, DRS restrictive eating frequency, and EDE weight and shape concern subscales. Separate models were run for each symptom, such that symptom‐specific predictors (e.g., duration and course of restrictive eating) were matched with symptom‐specific outcomes (e.g., DRS restrictive eating frequency). Follow‐up ANOVAs compared variance in symptom severity accounted for by each regression step. Benjamini–Hochberg corrections (Benjamini and Hochberg 1995) with a 5% false discovery rate corrected for multiple family‐wise comparisons.

## 3 Results

### 3.1 Participant Characteristics

Tables 1 and 2 detail sample demographic and clinical information. Most participants identified as cisgender female (94.7%) and White (93.3%), with at least some college education (93.3%). Ages ranged from 18 to 70 years, with a mean of 27.85 years (SD = 11.82). The AN group was significantly older than the AN‐WR group and more likely to report “1 ≥ years of college, no degree”. Mean baseline BMI in the full sample was 18.86 kg/m^2^ (SD = 1.76), reflecting the inclusion of both acute and weight‐restored participants. Across the sample, 65.3% of participants were enrolled in eating disorder treatment and 61.3% were taking psychotropic medications.

**TABLE 1:** Sample demographic characteristics for acute anorexia nervosa (AN) and weight‐restored AN (AN‐WR) at baseline.

Variables	Full sample (*n* = 75)	AN (*n* = 31)	AN‐WR (*n* = 44)
	Mean (SD) or %	Mean (SD) or %	Mean (SD) or %
Age (years)	27.85 (11.82)	35.41 (15.32)*	23.6 (6.23)*
Gender			
Cisgender male	2 (2.7%)	0 (0%)	2 (4.5%)
Cisgender female	71 (94.7%)	30 (96.7%)	41 (93.2%)
Transgender female	0 (0%)	0 (0%)	0 (0%)
Transgender male	0 (0%)	0 (0%)	0 (0%)
Other	2 (2.6%)	1 (3.2%)	1 (2.3%)
Race			
White	70 (93.3%)	30 (96.7%)	40 (90.9%)
African American/Black	0 (0%)	0 (0%)	0 (0%)
Native American	1 (1.3%)	0 (0%)	1 (2.3%)
Hispanic/Latino	2 (2.7%)	1 (3.2%)	1 (2.3%)
Asian American	0 (0%)	0 (0%)	0 (0%)
Other	2 (2.7%)	0 (0%)	2 (4.5%)
Current ED treatment			
(% yes)	49 (65.3%)	16 (59.3%)	33 (68.8%)
Outpatient individual therapy	44 (58.7%)	17 (54.8%)	27 (61.4%)
Outpatient group therapy	7 (9.3%)	3 (9.7%)	4 (9.1%)
Intensive outpatient therapy	3 (4.0%)	1 (3.2%)	2 (4.5%)
Nutritional counseling	17 (22.3%)	10 (32.3%)	7 (38.6%)
Medication management	16 (21.3%)	9 (29.0%)	7 (15.9%)
Other	1 (1.3%)	0 (0%)	1 (2.3%)
Psychiatric medication			
(% yes)	46 (61.3%)	20 (64.5%)	26 (59.1%)
Education			
High school graduate	5 (6.7%)	4 (12.9%)	1 (2.3%)
Some college, less than 1 year	9 (12%)	2 (6.5%)	7 (15.9%)
1 ≥ years of college, no degree	19 (25.3%)	3 (9.7%)*	16 (36.4%)*
Associate degree	8 (10.7%)	5 (16.1%)	3 (6.8%)
Bachelor's degree	23 (30.7%)	11 (35.5%)	12 (27.3%)
Master's degree	7 (9.3%)	4 (12.9%)	3 (6.8%)
Professional degree	0 (0%)	0 (0%)	0 (0%)
Doctorate degree	4 (5.3%)	2 (6.5%)	2 (4.5%)

Abbreviation: ED = eating disorder.

* Significant difference between AN and AN‐WR (*p* < 0.05).

**TABLE 2:** Sample clinical characteristics for acute anorexia nervosa (AN) and weight‐restored AN (AN‐WR).

Variables	Full sample (*n* = 75)	AN (*n* = 31)	AN‐WR (*n* = 44)
	Mean (SD)	Mean (SD)	Mean (SD)
BMI (kg/m^2^)			
Baseline	18.86 (1.76)	17.24 (1.26)*	20.00 (1.00)*
Follow‐up	20.33 (1.69)	—	20.33 (1.69)
DRS			
Baseline (days)	16.54 (10.81)	22.47 (7.76)*	12.50 (10.80)*
Follow‐Up (days)	9.41 (11.56)	—	9.41 (11.56)
EDE shape concern			
Baseline	3.23 (1.51)	3.55 (1.34)	3.00 (1.58)
Follow‐up	2.44 (1.78)	—	2.44 (1.78)
EDE weight concern			
Baseline	2.99 (1.64)	3.26 (1.65)	2.81 (1.62)
Follow‐up	2.10 (1.72)	—	2.10 (1.72)
General eating disorder			
Age of onset (years)	15.19 (4.74)	14.55 (5.02)	15.64 (4.54)
Duration (years)	11.49 (11.77)	17.03 (14.76)*	7.59 (6.99)*
Course	2.96 (1.03)	3.23 (0.99)	2.77 (1.03)
Low weight			
Age of onset (years)	16.85 (5.89)	15.68 (6.52)	17.68 (5.31)
Duration (years)	9.97 (13.15)	16.97 (16.56)*	5.05 (6.80)*
Course	2.19 (1.22)	2.55 (1.21)*	1.93 (1.17)*
Restrictive eating			
Age of onset (years)	15.69 (4.03)	15.90 (4.55)	15.55 (3.67)
Duration (years)	12.16 (11.58)	17.94 (14.21)*	8.09 (6.99)*
Course	2.73 (0.99)	2.97 (0.87)	2.57 (1.04)
Weight/shape preoccupation			
Age of onset (years)	13.13 (5.22)	12.87 (6.54)	13.32 (4.12)
Duration (years)	14.47 (12.02)	20.97 (14.06)*	9.89 (7.66)*
Course	3.11 (1.12)	3.39 (1.02)	2.91 (1.16)

Abbreviations: BMI = body mass index; DRS = Dietary Restriction Screener; EDE = Eating Disorder Examination.

* Significant difference in means between AN and AN‐WR (*p* < 0.05).

### 3.2 Duration and Course Descriptive Results

Eating disorder durations ranged from 0 to 49 years, with participants reporting, on average, just over a decade of illness (M = 11.49, SD = 11.77 years). As highlighted in Table 2, duration and course of weight/shape preoccupation was reported as the longest and most continuous symptom, followed by restrictive eating and then low weight. Notably, AN participants reported longer durations of their eating disorder and all eating disorder symptoms, as well as more chronic courses of underweight, primarily owing to their older age at baseline (Supporting Information Tables 2 and 3). All duration variables were highly correlated (*r* = 0.73–0.93), with restriction duration demonstrating the strongest association with general eating disorder duration (*r* = 0.91) (Supporting Information Table 4). Course variables were more modestly correlated (*r* = 0.11–0.52), with restriction course most strongly associated with general eating disorder course (*r* = 0.52). However, none of the illness or symptom duration measures was significantly correlated with any of the illness or symptom course measures, highlighting duration and course as distinct constructs.

### 3.3 Cross‐Sectional Regression Models

For all dependent variables, cross‐sectional models including symptom‐specific duration and course (Step 3) accounted for significantly more variance in symptom severity compared to models only including general illness duration and course (Step 2) (see Table 3).

**TABLE 3:** Cross‐sectional associations between general and symptom‐specific duration and course and eating disorder (ED) symptoms.

	Model 1: BMI			Model 2: restrictive eating			Model 3: shape concern			Model 4: weight concern		
	*B*	SE	*p*	*B*	SE	*p*	*B*	SE	*p*	*B*	SE	*p*
Step 1: covariates												
*R* ^2^_adj_	0.20			0.18			0.12			0.18		
*F*(*P*)	10.01 (< 0.001*)			8.96 (< 0.001*)			5.89 (0.004*)			9.01 (< 0.001*)		
Age	−0.07	0.02	< 0.001*	0.37	0.10	< 0.001*	0.02	0.01	0.087	0.04	0.01	0.011*
Current treatment	0.30	0.38	0.440	4.67	2.39	0.054	1.03	0.35	0.004*	1.23	0.36	0.001*
Step 2: general duration and course												
*R* ^2^_adj_	0.24			0.26			0.12			0.18		
*F*(*P*)	6.76 (< 0.001*)			7.52 (< 0.001*)			3.51 (0.011*)			4.90 (0.002*)		
Age	−0.05	0.03	0.036	0.27	0.15	0.082	0.00	0.02	0.932	0.03	0.02	0.294
Current treatment	0.58	0.39	0.143	2.48	2.37	0.298	0.92	0.36	0.013	1.10	0.38	0.005*
ED pattern	−0.41	0.19	0.032	3.29	1.13	0.005*	0.12	0.17	0.486	0.18	0.18	0.312
ED duration	−0.01	0.03	0.581	0.09	0.16	0.549	0.03	0.02	0.265	0.01	0.03	0.593
Step 3: symptom duration and course												
*R* ^2^_adj_	0.28			0.348			0.43			0.24		
*F*(*P*)	5.90 (< 0.001*)			7.48 (< 0.001*)			10.03 (< 0.001*)			4.85 (< 0.001*)		
Age	−0.03	0.04	0.484	0.50	0.28	0.080	−0.06	0.03	0.036	−0.01	0.03	0.763
Current treatment	0.42	0.39	0.283	2.53	2.24	0.262	0.87	0.29	0.004*	1.07	0.37	0.005*
ED pattern	−0.17	0.21	0.414	0.99	1.27	0.439	−0.09	0.14	0.524	0.07	0.18	0.715
ED duration	−0.02	0.03	0.518	0.37	0.25	0.140	0.00	0.02	0.951	0.00	0.03	0.898
Symptom pattern	−0.41	0.16	0.015	3.83	1.21	0.002*	0.65	0.13	< 0.001*	0.36	0.16	0.025
Symptom duration	−0.02	0.04	0.534	−0.51	0.44	0.251	0.09	0.03	0.009*	0.05	0.04	0.200

* Significant after Benjamini–Hochberg correction (*p* < 0.05).

#### 3.3.1 BMI

In Step 2, covariates and general illness duration and course accounted for 27.9% of the variance in baseline BMI (*R* ^2^ = 0.28, *F*(4,70) = 6.76, *p* < 0.001). An ANOVA comparing Steps 2 and 3 models found that adding duration and course of unhealthy low weight in Step 3 significantly improved the model (*p* = 0.043), increasing the variance explained by 6.4%. The Step 3 model was significant (*R* ^2^ = 0.34, *F*(6,68) = 5.90, *p* < 0.001). However, after familywise corrections, none of the general or symptom‐specific duration/course variables significantly predicted BMI in the final model.

#### 3.3.2 Restrictive Eating

In Step 2, covariates and general illness duration and course accounted for 30.4% of the variance in baseline restrictive eating (*R* ^2^ = 0.30, *F*(4,70) = 7.52, *p* < 0.001). An ANOVA comparing Step 2 and Step 3 models found that adding duration and course of restrictive eating in Step 3 significantly improved the model (*p* = 0.006), increasing variance explained by 9.7%. The Step 3 model was significant (*R* ^2^ = 0.40, *F*(6,68) = 7.48, *p* < 0.001). After familywise corrections, only a more continuous course of restrictive eating predicted more frequent baseline restrictive eating (*B* = 3.83, *p* = 0.002).

#### 3.3.3 Shape Concern

In Step 2, covariates and general illness duration and course accounted for 16.9% of the variance in baseline shape concern (*R* ^2^ = 0.17, *F*(4,70) = 3.52, *p* = 0.011). An ANOVA comparing the Step 2 and 3 models found that adding duration and course of weight/shape preoccupation in Step 3 significantly improved the model (*p* < 0.001), increasing variance explained by 30.4%. The Step 3 model was significant (*R* ^2^ = 0.47, *F*(6,68) = 10.03, *p* < 0.001). After familywise corrections, current treatment (*B* = 0.87, *p =* 0.004), longer weight/shape preoccupation duration (*B* = 0.09, *p* = 0.009), and more continuous course of weight/shape preoccupation (*B* = 0.65, *p* < 0.001) predicted higher baseline shape concern.

#### 3.3.4 Weight Concern

In Step 2, covariates and general illness duration and course accounted for 22.1% of the variance in baseline weight concern (*R* ^2^ = 0.22, *F*(4,70) = 4.90, *p* = 0.001). An ANOVA comparing Step 2 and Step 3 models found that adding duration and course of weight/shape preoccupation in Step 3 significantly improved the model (*p* = 0.025), increasing variance explained by 8.2%. The Step 3 model was significant (*R* ^2^ = 0.30, *F*(6,68) = 4.85, *p* < 0.001). However, after family‐wise correction, only current treatment predicted higher baseline weight concern (*B* = 1.07, *p* = 0.005).

### 3.4 Longitudinal Regression Models

Overall, Step 3 models were significant for all outcomes at the 12‐month follow‐up (see Table 4); however, none of the individual measures of general illness duration or course nor symptom duration or course predicted longitudinal symptom outcomes.

**TABLE 4:** Longitudinal prediction of eating disorder (ED) symptoms from general and symptom‐specific duration and course.

	Model 1: BMI			Model 2: restrictive eating			Model 3: shape concern			Model 4: weight concern		
	*B*	SE	*p*	*B*	SE	*p*	*B*	SE	*p*	*B*	SE	*p*
Step 1: covariates												
*R* ^2^_adj_	0.29			0.28			0.54			0.56		
*F*(*P*)	5.28 (0.005*)			5.07 (0.006*)			12.89 (< 0.001*)			13.91 (< 0.001*)		
Age	−0.07	0.04	0.127	0.62	0.31	0.055	0.04	0.04	0.294	0.04	0.04	0.256
Current treatment	−0.17	0.58	0.773	1.10	4.41	0.805	0.26	0.54	0.639	0.51	0.49	0.311
Baseline DV	0.70	0.28	0.017	0.35	0.20	0.084	0.76	0.15	< 0.001*	0.67	0.15	< 0.001*
Step 2: general duration and course												
*R* ^2^_adj_	0.32			0.31			0.51			0.53		
*F*(*p*)	3.86 (0.010*)			3.81 (0.010*)			7.54 (< 0.001*)			7.88 (< 0.001*)		
Age	−0.05	0.07	0.503	0.09	0.50	0.856	0.06	0.06	0.343	0.07	0.06	0.287
Current treatment	0.33	0.64	0.617	−0.15	4.51	0.974	0.09	0.60	0.887	0.52	0.55	0.353
Baseline DV	0.65	0.28	0.026	0.24	0.23	0.292	0.74	0.16	< 0.001*	0.67	0.16	< 0.001*
ED pattern	−0.50	0.29	0.099	2.83	2.33	0.236	0.17	0.27	0.546	−0.05	0.26	0.864
ED duration	−0.03	0.07	0.607	0.73	0.45	0.114	−0.02	0.06	0.728	−0.03	0.06	0.613
Step 3: symptom duration and course												
*R* ^2^_adj_	0.27			0.27			0.50			0.52		
*F*(*P*)	2.67 (0.034*)			2.65 (0.035*)			5.37 (< 001*)			5.76 (< 0.001*)		
Age	−0.05	0.07	0.513	0.03	0.55	0.963	0.08	0.09	0.405	0.12	0.09	0.162
Current treatment	0.33	0.67	0.629	−1.03	4.83	0.833	0.02	0.62	0.969	0.48	0.58	0.412
Baseline DV	0.72	0.30	0.027	0.244	0.25	0.341	0.61	0.21	0.007*	0.66	0.17	< 0.001*
ED pattern	−0.59	0.33	0.089	1.57	2.98	0.603	0.10	0.29	0.736	−0.18	0.29	0.533
ED duration	0.01	0.09	0.951	−0.24	1.48	0.874	−0.02	0.06	0.761	−0.02	0.06	0.722
Symptom pattern	0.13	0.28	0.632	1.10	2.43	0.653	0.31	0.30	0.316	0.29	0.24	0.237
Symptom duration	−0.04	0.07	0.572	1.00	1.47	0.503	0.00	0.07	0.974	−0.05	0.07	0.442

Abbreviation: DV = dependent variable (baseline value of DV examined in model).

* Significant after Benjamini–Hochberg correction (*α* < 0.05).

## 4 Discussion

Information about illness chronicity is often used to make important clinical decisions in the moment (e.g., about required treatment intensity) and regarding future prognosis. Therefore, we investigated the concurrent and predictive validity of different measures of illness chronicity on core clinical symptoms among individuals with AN. We found that regression models incorporating information about the duration and course of specific AN symptoms (i.e., low weight, restrictive eating, and weight/shape preoccupation) predicted baseline severity of the associated symptom above models only including general illness duration and course. Cross‐sectionally, a more continuous lifetime course of restrictive eating was associated with more frequent restrictive eating, and a longer duration and more continuous course of shape concern was associated with greater shape concern. However, none of the illness or symptom‐specific duration or course variables predicted symptom severity at 12‐month follow‐up in a subgroup of AN‐WR participants.

In line with our first hypothesis, symptom‐specific measures provided information about current symptom severity above measures of general illness alone. Although symptom‐specific measures were only significantly predictive of restrictive eating and shape concern after familywise corrections, trends towards the same pattern were found across all outcomes. General illness duration is traditionally used in research and clinical practice as an indicator of AN severity and prognosis (Broomfield et al. 2017). However, the mixed literature surrounding the relations between length of illness and clinical outcomes suggests that existing illness duration measures may be imprecise and lack predictive utility (Radunz et al. 2020). Because individuals are left to decide for themselves when their illness began, some may indicate onset as when cognitive symptoms began, whereas others may consider onset to commence with the occurrence of behavioral symptoms. In our sample, participants reported experiencing different clinical symptoms for different lengths of time, with illness onset most closely corresponding with restrictive eating onset. A substantial body of research suggests that symptom profile may vary among individuals with eating disorders depending on the order in which different symptoms began (Haiman and Devlin 1999; Reas and Grilo 2007; Yamamiya et al. 2023). Therefore, the duration and course of specific symptoms may provide information more precise and useful than that of general illness.

Another notable finding was that specific symptom course provided the most fruitful information about concurrent severity of the associated symptom. A more continuous course of restrictive eating was the only significant predictor of more frequent baseline restrictive eating, and a more continuous course of weight/shape preoccupation was a significant predictor of greater baseline shape concern (alongside symptom duration). This finding aligns with literature demonstrating that AN is associated with highly variable illness trajectories and symptom patterns. While some individuals with AN endure chronic and persistent illness courses, others experience intermittent symptom patterns; therefore, illness or symptom duration alone may be an incomplete marker of severity or risk (Radunz et al. 2020). For instance, continuously engaging in severe restriction for a shorter time period (e.g., 5 years) may be more problematic than discontinuously engaging in restriction for longer (e.g., 20 years, with only 2 total years of restriction and remission otherwise). In our sample, illness and symptom duration variables were not significantly correlated with illness and symptom course variables, suggesting that these represent independent facets of chronicity. Because symptom profiles change over time (Neumark‐Sztainer et al. 2010), clinicians and researchers may benefit from assessing the continuity of specific symptoms in addition to the more standard length of illness when assessing clinical severity. Even more granular assessments of symptom course (e.g., number of months or percentage of time with specific symptoms) may further differentiate levels of chronicity.

These results also highlight the utility of interrupting eating disorder symptoms and lengthening symptom‐free periods, even briefly, since more continuous symptom profiles were associated with higher baseline severity. This is consistent with prior investigations, in which even brief symptom interruptions predicted better long‐term outcomes in AN (Eddy et al. 2017). Further, our findings parallel research in addictive disorders demonstrating that longer remission between periods of relapse is indicative of better long‐term outcomes (Brecht and Herbeck 2014; Dennis et al. 2007). Similarly, some research suggests that individuals who relapse from an eating disorder more rapidly experience elevated clinical severity (Olmsted et al. 2015) and, conversely, those who respond to treatment more rapidly experience better outcomes (Linardon et al. 2016). One way in which rapid relapse may be harmful and rapid treatment response may be beneficial is through their influence on the relative length of symptom‐free periods in AN. Longer symptom‐free periods may provide a more extended opportunity to relearn healthy patterns related to eating and body image. However, because these findings are cross‐sectional, it is also possible that individuals with greater restrictive eating and poorer body image may struggle to maintain more continuous symptom‐free periods.

Finally, in contrast with our second hypothesis, neither the duration or course of general illness nor any specific symptom predicted 12‐month follow‐up outcomes in a subsample of AN‐WR participants. This finding, while preliminary due to the small follow‐up sample size and the inclusion of only AN‐WR participants, contrasts some previous studies that have found longer duration and greater chronicity of AN to predict poorer long‐term outcomes (Fichter et al. 2017). It is unclear if this result reflects a power issue due to the smaller sample size for the longitudinal analyses, differences in the sample composition of the AN‐WR participants who tended to be younger and have shorter durations and less severe courses of illness than the AN group, or a finding of clinical importance. Since illness and symptom durations and courses were retrospectively self‐reported at baseline, another possibility is that individuals with more severe baseline symptoms were biased to perceive their illness as more continuous. Variability in participant treatment enrollment and the relatively short 12‐month follow‐up period may have further limited the ability to detect longitudinal effects. However, this finding does align with literature suggesting that historical illness patterns in AN may not be effective predictors of relapse or treatment response (Sala et al. 2023; Wildes et al. 2017). Nonetheless, treatment decisions are being made based on the classification of “severe and enduring AN”, and while definitions of this group have varied, they often rely on measures of general illness duration and chronicity (Broomfield et al. 2017). Our study, among others, tentatively suggests that this construct may offer an incomplete understanding of how historical illness patterns relate to long‐term outcomes and that symptom improvement is possible for individuals with protracted AN illness lengths and courses (Eddy et al. 2017; Redgrave et al. 2021).

This study has several strengths, including the use of a novel measure that finely parsed illness duration and course metrics, a moderately‐sized sample representing different illness stages (e.g., acute and weight‐restored AN), and the ability to examine measures of illness duration and course as both cross‐sectional and longitudinal predictors of AN severity. However, results should be interpreted in the context of several limitations. First, this was an observational secondary analysis with a relatively homogeneous (i.e., majority White, cisgender female, college‐educated) sample. Barriers to care for groups underrepresented in eating disorder research and treatment (e.g., men, people of color) can cause symptoms to remain undiagnosed and untreated for longer (Penwell et al. 2024). As a result, illness course or duration may show different associations with symptom outcomes in these populations. Additionally, only AN‐WR participants completed follow‐up measures as the longitudinal data were only available for a parent study enrolling weight‐restored individuals. This may have impacted the power of the longitudinal analyses and their generalizability to acute AN. Further, measures of illness and symptom duration and course were all retrospectively self‐reported, introducing the possibility of recall bias and inaccuracy in reporting onset of certain symptoms (e.g., low weight). There is evidence that many individuals with AN under‐report symptoms (Vanzhula et al. 2024); therefore, duration and course of illness may have been more chronic and continuous than reported. Future studies would benefit from examining the predictive validity of illness and symptom chronicity in a larger, more diverse sample with both acute and weight‐restored AN, more thoroughly assessing illness course and treatment status, and using more objective measures (e.g., documented onset of low weight through medical records) and in‐the‐moment symptom measures (e.g., ecological momentary assessment).

In conclusion, our results suggest that researchers and clinicians may benefit from expanding beyond assessing general illness duration to assess for chronicity of specific symptoms and the continuity of symptom course. However, we also found preliminary evidence that historical illness patterns did not predict long‐term outcomes for those with weight‐restored AN. These results may offer hope to individuals with long‐standing illness that their past illness patterns may not influence their ability to pursue recovery.

## Author Contributions

**Kira G. Venables:** conceptualization, writing – original draft, visualization, writing – review and editing, formal analysis, data curation. **Ariana R. Bazzi:** writing – original draft, writing – review and editing. **Abigail Smith:** writing – original draft, writing – review and editing. **Soo‐Eun Lee:** visualization, writing – review and editing. **Carol B. Peterson:** investigation, funding acquisition, writing – review and editing, supervision. **Ann F. Haynos:** conceptualization, investigation, funding acquisition, writing – original draft, writing – review and editing, data curation, supervision.

## Conflicts of Interest

The authors declare no conflicts of interest.

## Supporting information


**Data S1:** eat24525‐sup‐0001‐Supinfo.docx.

## Data Availability

The data that support the findings of this study are available from the corresponding author upon reasonable request.

## References

[eat24525-bib-0001] Ålgars, M. , S. Oshukova , and J. Suokas . 2023. “A Novel Outpatient Treatment Model for Patients With Severe and Enduring Anorexia Nervosa: An Observational Study of Patient Characteristics, Treatment Goals, and Treatment Course.” Journal of Eating Disorders 11: 1–9. 10.1186/s40337-023-00877-x. 37674214 PMC10481592

[eat24525-bib-0002] Bardone‐Cone, A. M. , R. A. Hunt , and H. J. Watson . 2018. “An Overview of Conceptualizations of Eating Disorder Recovery, Recent Findings, and Future Directions.” Current Psychiatry Reports 20: 79. 10.1007/s11920-018-0932-9. 30094740

[eat24525-bib-0004] Benjamini, Y. , and Y. Hochberg . 1995. “Controlling the False Discovery Rate: A Practical and Powerful Approach to Multiple Testing.” Journal of the Royal Statistical Society, Series B: Statistical Methodology 57: 289–300. 10.1111/j.2517-6161.1995.tb02031.x.

[eat24525-bib-0005] Berg, K. C. , C. B. Peterson , P. Frazier , and S. J. Crow . 2012. “Psychometric Evaluation of the Eating Disorder Examination and Eating Disorder Examination‐Questionnaire: A Systematic Review of the Literature.” International Journal of Eating Disorders 45: 428–438. 10.1002/eat.20931. 21744375 PMC3668855

[eat24525-bib-0006] Brecht, M. L. , and D. Herbeck . 2014. “Time to Relapse Following Treatment for Methamphetamine Use: A Long‐Term Perspective on Patterns and Predictors.” Drug and Alcohol Dependence 139: 18–25. 10.1016/j.drugalcdep.2014.02.702. 24685563 PMC4550209

[eat24525-bib-0007] Broomfield, C. , K. Stedal , S. Touyz , and P. Rhodes . 2017. “Labeling and Defining Severe and Enduring Anorexia Nervosa: A Systematic Review and Critical Analysis.” International Journal of Eating Disorders 50, no. 6: 611–623. 10.1002/eat.22715. 28444828

[eat24525-bib-0008] Carter, J. C. , K. B. Mercer‐Lynn , S. J. Norwood , et al. 2012. “A Prospective Study of Predictors of Relapse in Anorexia Nervosa: Implications for Relapse Prevention.” Psychiatry Research 200, no. 2–3: 518–523. 10.1016/j.psychres.2012.04.037. 22657951

[eat24525-bib-0010] Cooper, Z. , and C. Fairburn . 1987. “The Eating Disorder Examination: A Semi‐Structured Interview for the Assessment of the Specific Psychopathology of Eating Disorders.” International Journal of Eating Disorders 6, no. 1: 1–8.

[eat24525-bib-0011] Davis, L. , B. T. Walsh , J. Schebendach , D. R. Glasofer , and J. E. Steinglass . 2020. “Habits Are Stronger With Longer Duration of Illness and Greater Severity in Anorexia Nervosa.” International Journal of Eating Disorders 53, no. 5: 683–689. 10.1002/eat.23265. PMC7217727 32227516

[eat24525-bib-0012] Dennis, M. L. , M. A. Foss , and C. K. Scott . 2007. “An Eight‐Year Perspective on the Relationship Between the Duration of Abstinence and Other Aspects of Recovery.” Evaluation Review 31, no. 6: 585–612. 10.1177/0193841X07307771. 17986709

[eat24525-bib-0013] Eddy, K. T. , N. Tabri , J. J. Thomas , et al. 2017. “Recovery From Anorexia Nervosa and Bulimia Nervosa at 22‐Year Follow‐Up.” Journal of Clinical Psychiatry 78, no. 2: 184–189. 10.4088/JCP.15m10393. 28002660 PMC7883487

[eat24525-bib-0014] Eielsen, H. P. , K. Vrabel , A. Hoffart , Ø. Rø , and J. H. Rosenvinge . 2022. “Reciprocal Relationships Between Personality Disorders and Eating Disorders in a Prospective 17‐Year Follow‐Up Study.” International Journal of Eating Disorders 55, no. 12: 1753–1764. 10.1002/eat.23823. 36214278 PMC10092669

[eat24525-bib-0016] Fernández‐Aranda, F. , J. Treasure , G. Paslakis , et al. 2021. “The Impact of Duration of Illness on Treatment Nonresponse and Drop‐Out: Exploring the Relevance of Enduring Eating Disorder Concept.” European Eating Disorders Review 29: 499–513. 10.1002/erv.2822. 33599348

[eat24525-bib-0017] Fichter, M. , and N. Quadflieg . 2001. “The Structured Interview for Anorexic and Bulimic Disorders for DSM‐IV and ICD‐10 (SIAB‐EX): Reliability and Validity.” European Psychiatry 16, no. 1: 38–48. 10.1016/s0924-9338(00)00534-4. 11246291

[eat24525-bib-0018] Fichter, M. M. , S. Herpertz , N. Quadflieg , and B. Herpertz‐Dahlmann . 1998. “Structured Interview for Anorexic and Bulimic Disorders for DSM‐IV and ICD‐10: Updated (Third) Revision.” International Journal of Eating Disorders 24, no. 3: 227–249. 10.1002/(sici)1098-108x(199811)24:3<227::aid-eat1>3.0.co;2-o. 9741034

[eat24525-bib-0019] Fichter, M. M. , N. Quadflieg , R. D. Crosby , and S. Koch . 2017. “Long‐Term Outcome of Anorexia Nervosa: Results From a Large Clinical Longitudinal Study.” International Journal of Eating Disorders 50, no. 9: 1018–1030. 10.1002/eat.22736. 28644530

[eat24525-bib-0020] Fichter, M. M. , N. Quadflieg , and S. Hedlund . 2006. “Twelve‐Year Course and Outcome Predictors of Anorexia Nervosa.” International Journal of Eating Disorders 39: 87–100. 10.1002/eat.20215. 16231345

[eat24525-bib-0021] First, M. B. , J. B. W. Williams , R. S. Karg , and R. L. Spitzer . 2015. Structured Clinical Interview for DSM‐5‐Research Version (SCID‐5 for DSM‐5, Research Version; SCID‐5‐RV). American Psychiatric Association.

[eat24525-bib-0022] Gaudiani, J. L. , A. Bogetz , and J. Yager . 2022. “Terminal Anorexia Nervosa: Three Cases and Proposed Clinical Characteristics.” Journal of Eating Disorders 10, no. 1: 23. 10.1186/s40337-022-00548-3. 35168671 PMC8845309

[eat24525-bib-0023] Glasofer, D. R. , A. F. Muratore , E. Attia , et al. 2020. “Predictors of Illness Course and Health Maintenance Following Inpatient Treatment Among Patients With Anorexia Nervosa.” Journal of Eating Disorders 8: 1–10. 10.1186/s40337-020-00348-7. 33292619 PMC7709230

[eat24525-bib-0024] Haiman, C. , and M. J. Devlin . 1999. “Binge Eating Before the Onset of Dieting: A Distinct Subgroup of Bulimia Nervosa?” International Journal of Eating Disorders 25, no. 2: 151–157. 10.1002/(sici)1098-108x(199903)25:2<151::aid-eat4>3.0.co;2-5. 10065392

[eat24525-bib-0025] Haynos, A. F. , and A. E. Fruzzetti . 2015. “Initial Evaluation of a Single‐Item Screener to Assess Problematic Dietary Restriction.” Eating and Weight Disorders 20, no. 3: 405–413. 10.1007/s40519-014-0161-0. 25412874 PMC5904791

[eat24525-bib-0026] Haynos, A. F. , and C. A. Roberto . 2017. “The Effects of Restaurant Menu Calorie Labeling on Hypothetical Meal Choices of Females With Disordered Eating.” International Journal of Eating Disorders 50, no. 3: 275–283. 10.1002/eat.22675. 28130796 PMC5378635

[eat24525-bib-0027] Keel, P. K. , and T. A. Brown . 2010. “Update on Course and Outcome in Eating Disorders.” International Journal of Eating Disorders 43: 195–204. 10.1002/eat.20810. 20186717

[eat24525-bib-0028] Khalsa, S. S. , L. C. Portnoff , D. McCurdy‐McKinnon , and J. D. Feusner . 2017. “What Happens After Treatment? A Systematic Review of Relapse, Remission, and Recovery in Anorexia Nervosa.” Journal of Eating Disorders 5: 20. 10.1186/s40337-017-0145-3. 28630708 PMC5470198

[eat24525-bib-0029] Linardon, J. , L. Brennan , and X. de la Piedad Garcia . 2016. “Rapid Response to Eating Disorder Treatment: A Systematic Review and Meta‐Analysis.” International Journal of Eating Disorders 49, no. 10: 905–919. 10.1002/eat.22595. 27528478

[eat24525-bib-0030] Marcolini, F. , A. Ravaglia , S. Tempia Valenta , et al. 2024. “Severe Enduring Anorexia Nervosa (SE‐AN) Treatment Options and Their Effectiveness: A Review of Literature.” Journal of Eating Disorders 12, no. 1: 48. 10.1186/s40337-024-01006-y. 38654374 PMC11040982

[eat24525-bib-0031] Meule, A. , D. R. Kolar , E. Rauh , and U. Voderholzer . 2023. “Comparing Illness Duration and Age as Predictors of Treatment Outcome in Female Inpatients With Anorexia Nervosa.” Eating Disorders 31, no. 3: 274–284. 10.1080/10640266.2022.2114586. 36178330

[eat24525-bib-0032] Neumark‐Sztainer, D. , N. I. Larson , J. A. Fulkerson , M. E. Eisenberg , and M. Story . 2010. “Family Meals and Adolescents: What Have We Learned From Project EAT (Eating Among Teens)?” Public Health Nutrition 13, no. 7: 1113–1121. 10.1017/S1368980010000169. 20144257

[eat24525-bib-0033] Olmsted, M. P. , D. E. MacDonald , T. McFarlane , K. Trottier , and P. Colton . 2015. “Predictors of Rapid Relapse in Bulimia Nervosa.” International Journal of Eating Disorders 48, no. 3: 337–340. 10.1002/eat.22380. 25545720

[eat24525-bib-0034] Penwell, T. E. , S. P. Bedard , R. Eyre , and C. A. Levinson . 2024. “Eating Disorder Treatment Access in the United States: Perceived Inequities Among Treatment Seekers.” Psychiatric Services 75, no. 10: 944–952. 10.1176/appi.ps.20230193. 38716514

[eat24525-bib-0035] Puckett, L. , D. Grayeb , V. Khatri , K. Cass , and P. Mehler . 2021. “A Comprehensive Review of Complications and New Findings Associated With Anorexia Nervosa.” Journal of Clinical Medicine 10, no. 12: 2555. 10.3390/jcm10122555. 34207744 PMC8226688

[eat24525-bib-0036] Radunz, M. , E. Keegan , I. Osenk , and T. D. Wade . 2020. “Relationship Between Eating Disorder Duration and Treatment Outcome: Systematic Review and Meta‐Analysis.” International Journal of Eating Disorders 53, no. 11: 1761–1773. 10.1002/eat.23373. 32856329

[eat24525-bib-0037] Ranzenhofer, L. M. , M. Jablonski , L. Davis , J. Posner , B. T. Walsh , and J. E. Steinglass . 2022. “Early Course of Symptom Development in Anorexia Nervosa.” Journal of Adolescent Health 71, no. 5: 587–593. 10.1016/j.jadohealth.2022.06.010 PMC10375485 35973892

[eat24525-bib-0038] Raykos, B. C. , D. M. Erceg‐Hurn , P. M. McEvoy , A. Fursland , and G. Waller . 2018. “Severe and Enduring Anorexia Nervosa? Illness Severity and Duration Are Unrelated to Outcomes From Cognitive Behaviour Therapy.” Journal of Consulting and Clinical Psychology 86, no. 8: 702–709. 10.1037/ccp0000319. 30035586

[eat24525-bib-0039] Reas, D. L. , and C. M. Grilo . 2007. “Timing and Sequence of the Onset of Overweight, Dieting, and Binge Eating in Overweight Patients With Binge Eating Disorder.” International Journal of Eating Disorders 40, no. 2: 165–170. 10.1002/eat.20353. 17089414

[eat24525-bib-0040] Redgrave, G. W. , C. C. Schreyer , J. W. Coughlin , L. K. Fischer , A. Pletch , and A. S. Guarda . 2021. “Discharge Body Mass Index, Not Illness Chronicity, Predicts 6‐Month Weight Outcome in Patients Hospitalized With Anorexia Nervosa.” Frontiers in Psychiatry 12: 641861. 10.3389/fpsyt.2021.641861. 33716836 PMC7946839

[eat24525-bib-0041] Romano, K. A. , K. E. Heron , R. Amerson , L. M. Howard , R. I. MacIntyre , and T. B. Mason . 2020. “Changes in Disordered Eating Behaviors Over 10 or More Years: A Meta‐Analysis.” International Journal of Eating Disorders 53, no. 7: 1034–1055. 10.1002/eat.23288. 32415907

[eat24525-bib-0042] Sala, M. , A. Keshishian , S. Song , et al. 2023. “Predictors of Relapse in Eating Disorders: A Meta‐Analysis.” Journal of Psychiatric Research 158: 281–299. 10.1016/j.jpsychires.2023.01.002. 36623362

[eat24525-bib-0043] Sheehan, D. V. , Y. Lecrubier , K. H. Sheehan , et al. 1998. “The Mini‐International Neuropsychiatric Interview (M.I.N.I.): The Development and Validation of a Structured Diagnostic Psychiatric Interview for DSM‐IV and ICD‐10.” Journal of Clinical Psychiatry 59, no. Suppl 20: 22–57. 9881538

[eat24525-bib-0054] Solmi, M. , J. Radua , M. Olivola , et al. 2021. “Age at Onset of Mental Disorders Worldwide: Large‐Scale Meta‐Analysis of 192 Epidemiological Studies.” Molecular Psychiatry 27, no. 1: 281–295. 10.1038/s41380-021-01161-7. 34079068 PMC8960395

[eat24525-bib-0044] Takakura, S. , C. S. Aso , K. Toda , T. Hata , M. Yamashita , and N. Sudo . 2019. “Physical and Psychological Aspects of Anorexia Nervosa Based on Duration of Illness: A Cross‐Sectional Study.” BioPsychoSocial Medicine 13: 1–7. 10.1186/s13030-019-0173-0. 31889996 PMC6929428

[eat24525-bib-0045] Touyz, S. , and P. Hay . 2015. “Severe and Enduring Anorexia Nervosa (SE‐AN): In Search of a New Paradigm.” Journal of Eating Disorders 3: 26. 10.1186/s40337-015-0065-z. 26236477 PMC4521346

[eat24525-bib-0046] Vanzhula, I. , K. Hagan , S. A. Duck , et al. 2024. “Eating Disorder Symptom Non‐Endorsers in Hospitalised Patients With Anorexia Nervosa: Who Are They?” European Eating Disorders Review 32, no. 4: 795–808. 10.1002/erv.3087. 38528330

[eat24525-bib-0047] Wang, S. B. , K. R. Fox , C. Boccagno , et al. 2021. “Functional Assessment of Restrictive Eating: A Three‐Study Clinically Heterogeneous and Transdiagnostic Investigation.” Journal of Abnormal Psychology 130, no. 7: 761–774. 10.1037/abn0000700. 34780230 PMC8597895

[eat24525-bib-0048] Wang, S. B. , E. M. Pisetsky , J. M. Skutch , A. E. Fruzzetti , and A. F. Haynos . 2018. “Restrictive Eating and Nonsuicidal Self‐Injury in a Nonclinical Sample: Co‐Occurrence and Associations With Emotion Dysregulation and Interpersonal Problems.” Comprehensive Psychiatry 82: 128–132. 10.1016/j.comppsych.2018.02.005. 29477705 PMC6167742

[eat24525-bib-0049] Webb, H. , B. Dalton , M. Irish , et al. 2022. “Clinicians' Perspectives on Supporting Individuals With Severe Anorexia Nervosa in Specialist Eating Disorder Intensive Treatment Settings.” Journal of Eating Disorders 10: 1–13. 10.1186/s40337-021-00528-z. 34991715 PMC8733908

[eat24525-bib-0050] Wildes, J. E. , K. T. Forbush , K. E. Hagan , et al. 2017. “Characterizing Severe and Enduring Anorexia Nervosa: An Empirical Approach.” International Journal of Eating Disorders 50, no. 4: 389–397. 10.1002/eat.22651. 27991694 PMC5386793

[eat24525-bib-0051] Wonderlich, S. A. , C. M. Bulik , U. Schmidt , H. Steiger , and H. W. Hoek . 2020. “Severe and Enduring Anorexia Nervosa: Update and Observations About the Current Clinical Reality.” International Journal of Eating Disorders 53, no. 8: 1303–1312. 10.1002/eat.23283. 32359125

[eat24525-bib-0052] Yamamiya, Y. , C. D. Desjardins , and E. Stice . 2023. “Sequencing of Symptom Emergence in Anorexia Nervosa, Bulimia Nervosa, Binge Eating Disorder, and Purging Disorder in Adolescent Girls and Relations of Prodromal Symptoms to Future Onset of These Eating Disorders.” Psychological Medicine 53, no. 10: 4657–4665. 10.1017/S0033291722001568. 37698515

