# Supplementary material for: General and Symptom‐Specific Illness Duration and Course as Predictors of Symptom Severity in Anorexia Nervosa
Source: Int J Eat Disord. 2025 Oct 2;59(1):99–110. doi: 10.1002/eat.24525 (PMC12773668; doi:10.1002/eat.24525)
Supplement: Supplementary file 1 — Data S1: eat24525‐sup‐0001‐Supinfo.docx. [file EAT-59-99-s001.docx]

**Course/Duration of Illness in AN Supplement**

**A. Parent Study Inclusion and Exclusion Criteria**

*Study 1. Neural Correlates of Reward in Anorexia Nervosa*

Participants from this dataset in the current analysis: *n* = 36 anorexia nervosa, weight-restored (AN-WR). This study examined reward and decision-making metrics in AN-WR participants and how deficits in these metrics predict relapse. Participants in the parent study were adults (*N* = 65) with AN-WR (*n* = 36) or matched healthy controls (HC; *n* = 29). At baseline, participants completed interviews, questionnaires, fMRI scanning, decision-making tasks completed both inside and outside of the scanner, and a multi-item test meal. At follow up, the AN-WR group completed clinical interviews at 3, 6, 9, and 12 months and questionnaires at 6 and 12 months.

For both the AN-WR and HC groups, inclusion criteria were: 1) age ≥ 18 years old, 2) current BMI ≥ 18.5 kg/m^2^, 3) ability to read and speak in English, and 4) right-handed. For the AN-WR group only, additional inclusion criteria were 1) DSM-5 diagnosis of AN in the past 12 months (established by the SCID-5-RV), with the exception of body image disturbance and intense fear of weight gain while underweight if they were actively engaging in weight control behavior, in line with prior research (Engel et al., 2013). This criterion was designed to capture those with AN who may underreport or under-endorse body image concern, given that some studies have suggested that up to one-third of individuals with AN may not report cognitive symptoms or may report "non-fat-phobic AN" (Becker et al., 2009; Vanzhula et al., 2024).

For both the AN-WR and HC groups, exclusion criteria were: 1) medical instability or current pregnancy, 2) current substance use disorder, psychosis, or bipolar-I disorder, 3) acute suicidality, 4) contraindication for fMRI (e.g., implanted metal), 5) history of neurological disorder/injury (e.g., stroke; head injury with > 10 minutes loss of consciousness), 6) food allergy that cannot be accommodated through substitutions to the laboratory test meal, and 7) lacking capacity to consent. For the HC group only, additional exclusion criteria were: 1) current DSM-5 Axis-I diagnosis or current or past eating disorder diagnosis (established by the SCID-5-RV) and 2) current use of psychotropic medication.

*Study 2. Real-time fMRI Neurofeedback to Alter Limbic Disturbances in Anorexia Nervosa*

Participants from this dataset in the current analysis: *n* = 2 acute AN. This pilot study examined whether real-time functional magnetic resonance imaging (fMRI) neurofeedback improved emotion regulation and eating disorder symptoms in AN. Participants in the parent study were adults with acute AN (*N* = 11). At baseline, participants completed interviews and questionnaires. Eligible participants completed an emotion regulation task during fMRI scanning, with (*n* = 6) or without (*n* = 5) real-time fMRI neurofeedback.

Inclusion criteria were: 1) DSM-5 diagnosis of AN, with the exception of body image disturbance and intense fear of weight gain (established by the MINI) if underweight and actively engaging in weight-control behavior, 2) age ≥ 18 years old, 3) ability to read and speak in English, and 4) right-handed. Exclusion criteria were 1) Medical instability or current pregnancy (self-reported), 2) acute suicidality, current substance use disorder, psychosis, or mania, 3) contraindication for fMRI (e.g., implanted metal), and 4) history of neurological disorder/injury (e.g., stroke; head injury with > 10 minutes loss of consciousness).

*Study 3. Real-time fMRI Neurofeedback versus Sham in Anorexia Nervosa.*

Participants from this dataset in the current analysis: *n* = 5 acute AN and *n* = 8 AN-WR. This randomized study compared real-time fMRI neurofeedback to sham neurofeedback for improving emotion regulation and eating disorder symptoms in AN and AN-WR. Participants in the parent study were adults with AN or AN-WR (*N* = 23). At baseline, participants completed interviews and questionnaires. Eligible participants were randomized to receive either real-time neurofeedback (*n* = 10) or a sham condition (*n* = 13) while undergoing fMRI scanning with an emotion regulation task during one to two sessions. Participants then completed online follow-up assessments on changes in affective and eating disorder symptoms approximately one week after their final neurofeedback session.

Inclusion criteria were 1) DSM-5 diagnosis of AN within the last 12 months (established by the MINI), with the exception of body image disturbance and intense fear of weight gain while underweight if actively engaging in weight-control behavior, 2) age ≥ 18 years old, 3) ability to read and speak in English, and 4) right-handed. Exclusion criteria were 1) Medical instability or current pregnancy (self-reported), 2) acute suicidality, current substance use disorder, psychosis, or mania, 3) contraindication for fMRI (e.g., implanted metal), and 4) history of neurological disorder/injury (e.g., stroke; head injury with > 10 minutes loss of consciousness).

*Study 4. Positive Affect Treatment (PAT) to Target Reward Mechanisms in Anorexia Nervosa.*

Participants from this dataset in the current analysis: *n* = 20 acute AN. This study investigated the efficacy of an adaptation of Positive Affect Treatment for Anorexia Nervosa (PAT-AN) to alter reward responsivity and eating disorder symptoms in AN. Participants in the parent study were adults with AN (*N* = 20) randomized to receive 20 weeks of PAT-AN (*n* = 10) or waitlist (*n* = 10). All participants completed baseline assessments. PAT-AN participants completed weekly weight and symptom measures and repeated the assessment battery at post-treatment and 3-month follow-up. Waitlist participants completed their second assessment visit approximately 20 weeks after baseline and were given the option to receive PAT-AN thereafter.

Inclusion criteria were 1) age ≥ 18 years old, 2) DSM-5 diagnosis of AN (established by the adapted MINI), 3) ability to read and speak in English, and 4) involvement in ongoing oversight with a primary physical or mental health provider as defined by: a) identification by the participant of a physical or mental health provider (e.g., physician, psychiatrist, psychologist, masters level social worker, licensed professional clinical counselor, licensed marriage and family therapist) who will serve as the primary provider throughout the study; and b) willingness to sign a Release of Information that gives the study therapist the right to discuss any change in medical or psychiatric stability or other health concerns with the participant’s identified primary provider. Exclusion criteria were 1) medical instability according to standard guidelines and medical staff judgment, 2) acute suicidality, current substance use disorder, psychosis, or mania requiring specialized treatment, 3) lacking capacity to consent, and 4) current pregnancy (self-reported).

*Study 5. Investigating the Impact of Oxytocin on the Neurobiological Underpinnings of Socioemotional Deficits in Anorexia Nervosa.*

Participants from this dataset in the current analysis: *n* = 4 acute AN. This study examined the use of intranasal oxytocin to alter emotion regulation deficits in AN compared to HC. In the parent study, participants with AN (*n* = 8) and matched healthy controls (HC; *n* = 6) completed interviews and questionnaires at baseline, followed by two visits approximately one week apart: one visit with INOT and one with placebo, administered double blind. After receiving the dose of INOT or placebo, participants underwent an fMRI scan (with two socioemotional tasks) and a single item test meal.

For both the AN and HC groups, inclusion criteria were 1) age ≥ 18 years old, 2) female, 3) ability to read and speak in English, and 4) right-handed. For the AN group, additional inclusion criteria were 1) DSM-5 diagnosis of AN (established by the SCID-5-RV). For both the AN and HC groups, exclusion criteria were 1) medical instability or current pregnancy or lactation, 2) current substance use disorder, psychosis, or mania, 3) contraindication for fMRI (e.g., implanted metal), 4) history of neurological disorder/injury (e.g., stroke; head injury with > 10 minutes loss of consciousness), 5) food allergy that cannot be accommodated through substitutions to the laboratory test meal, 6) lacking capacity to consent, 7) contraindications for INOT administration, and 8) acute suicidality. For the HC group only, additional exclusion criteria were 1) current DSM-5 Axis-I diagnosis or current or past eating disorder diagnosis (established by the SCID-5-RV and EDE), 2) BMI < 19.0, and 3) current use of psychoactive medications (e.g., antidepressants, antipsychotics). For the AN group only, additional exclusion criteria were 1) psychoactive medication change within the past 6 weeks.

**B. Course of Illness Questionnaire**

We would like to ask you a few questions to understand your lifetime pattern of disordered eating.

1. Have you ever had an eating disorder? **YES NO**

**IF YES:**

2. At what age do you think you first developed an eating disorder? ______

3. Working back from today, how long has it been since you had an eating disorder (please answer in weeks)? If you still have an eating disorder, write zero _____

4. From the time that you when first developed an eating disorder until now, please choose the pattern that BEST describes the course of your eating disorder. For this question, don’t worry about the specific type of eating disorder. We are just trying to understand the overall pattern of your eating disorder.

0 = No eating disorder.


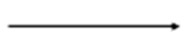


1 = Short episodes of eating disorder symptoms followed by longer intervals without eating disorder symptoms. Overall, eating disorder symptoms have been present 30% of the time or less.


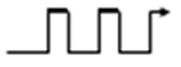


2 = Periods of eating disorder symptoms and periods without of approximate length. Overall, eating disorder symptoms have been present about 30-60% of the time.


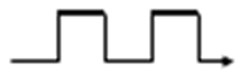


3 = Long periods of eating disorder symptoms with only short intervals without eating disorder symptoms. Overall, eating disorder symptoms have been present 60-90% of the time.


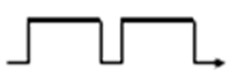


4 = Chronic eating disorder symptoms. Eating disorder symptoms have been present about 90-100% of the time.


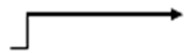


5. What is your lowest adult weight? ______

6. What is your highest lifetime weight (excluding pregnancy)? ______

7. Have you ever been at an **unhealthy low weight**? **YES NO**

**IF YES:**

8. At what age do you think you first were at an **unhealthy low weight**? ______

9. Working back from today, how long has it been since you were at an **unhealthy low weight** (please answer in weeks)? If you still are at an unhealthy low weight, write zero ______

10. From the time that you first were at an **unhealthy low weight** until now, please choose the pattern that BEST describes your pattern of **unhealthy low weight**.

0 = No unhealthy low weight.


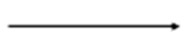


1 = Short episodes of unhealthy low weight followed by longer intervals without unhealthy low weight. Overall, unhealthy low weight has been present 30% of the time or less.


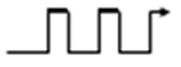


2 = Periods of unhealthy low weight and periods without of approximate length. Overall, unhealthy low weight has been present about 30-60% of the time.


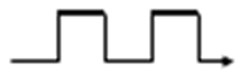


3 = Long periods of unhealthy low weight with only short intervals without unhealthy low weight. Overall, unhealthy low weight has been present 60-90% of the time.


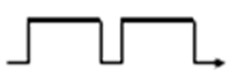


4 = Chronic unhealthy low weight. Unhealthy low weight has been present about 90-100% of the time. There have been no major intervals without unhealthy low weight.


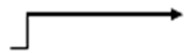


11. Have you ever engaged in **restrictive eating** (eating less food than is healthy for your body)?

**IF YES:**

12. At what age did you first engage in **restrictive eating**? ______

13. Working back from today, how long has it been since you have engaged in **restrictive eating** (please answer in weeks)? If you have engaged in restrictive eating this week, write zero ___

14. From the time that you first engaged in **restrictive eating** (eating less food than is healthy for your body) until now, please choose the pattern that BEST describes your pattern of **restrictive eating**.

0 = No restrictive eating.


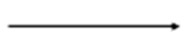


1 = Short episodes of restrictive eating followed by longer intervals without restrictive eating. Overall, restrictive eating has been present 30% of the time or less.


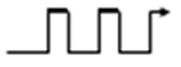


2 = Periods of restrictive eating and periods without of approximate length. Overall, restrictive eating has been present about 30-60% of the time.


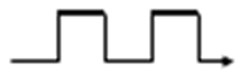


3 = Long periods of restrictive eating with only short intervals without restrictive eating. Overall, restrictive eating has been present 60-90% of the time.


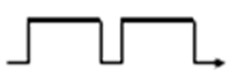


4 = Chronic restrictive eating. Restrictive eating has been present about 90-100% of the time. There have been no major intervals without restrictive eating.


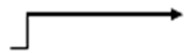


15. Have you ever engaged in binge eating (eating a large amount of food in a short period of time while feeling out of control)? **YES NO**

**IF YES:**

16. At what age did you first engage in **binge eating**? ______

17. Working back from today, how long has it been since you have engaged in **binge eating** (please answer in weeks)? If you have engaged in binge eating this week, write zero ___

18. From the time that you first engaged in **binge eating** (eating a large amount of food in a short period of time while feeling out of control) until now, please choose the pattern that BEST describes your pattern of **binge eating**.

0 = No binge eating.


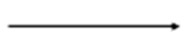


1 = Short episodes of binge eating followed by longer intervals without binge eating. Overall, binge eating has been present 30% of the time or less.


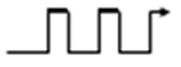


2 = Periods of binge eating and periods without of approximate length. Overall, binge eating has been present about 30-60% of the time.


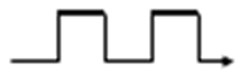


3 = Long periods of binge eating with only short intervals without binge eating. Overall, binge eating has been present 60-90% of the time.


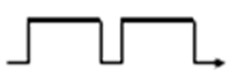


4 = Chronic binge eating. Binge eating has been present about 90-100% of the time. There have been no major intervals without binge eating.


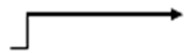


19. Have you ever engaged in self-induced vomiting? **YES NO**

**IF YES:**

20. At what age did you first engage in **self-induced vomiting**? ______

21. Working back from today, how long has it been since you have engaged in **self-induced vomiting** (please answer in weeks)? If you have engaged in self-induced vomiting this week, write zero ___

22. From the time that you first engaged in **self-induced vomiting** until now, please choose the pattern that BEST describes your pattern of **self-induced vomiting**.

0 = No self-induced vomiting.


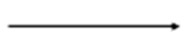


1 = Short episodes of self-induced vomiting followed by longer intervals without self-induced vomiting. Overall, self-induced vomiting has been present 30% of the time or less.


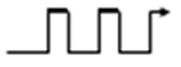


2 = Periods of self-induced vomiting and periods without of approximate length. Overall, self-induced vomiting has been present about 30-60% of the time.


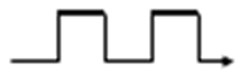


3 = Long periods of self-induced vomiting with only short intervals without self-induced vomiting. Overall, self-induced vomiting has been present 60-90% of the time.


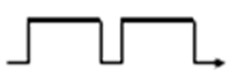


4 = Chronic self-induced vomiting. Self-induced vomiting has been present about 90-100% of the time. There have been no major intervals without self-induced vomiting.


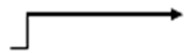


23. Have you ever engaged in **laxative use** for reasons pertaining to weight and shape?

**YES NO**

**IF YES:**

24. At what age did you first engage in **laxative use** for reasons pertaining to weight and shape? ______

25. Working back from today, how long has it been since you have engaged in **laxative use** for reasons pertaining to weight and shape? (please answer in weeks)? If you have engaged in laxative use this week, write zero ______

26. From the time that you first engaged in **laxative use** until now, please choose the pattern that BEST describes your pattern of **laxative use** for reasons pertaining to weight and shape.

0 = No laxative use.


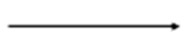


1 = Short episodes of laxative use followed by longer intervals without laxative use. Overall, laxative use has been present 30% of the time or less.


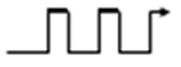


2 = Periods of laxative use and periods without of approximate length. Overall, laxative use has been present about 30-60% of the time.


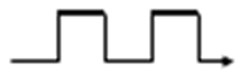


3 = Long periods of laxative use with only short intervals without laxative use. Overall, laxative use has been present 60-90% of the time.


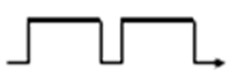


4 = Chronic laxative use. Laxative use has been present about 90-100% of the time. There have been no major intervals without laxative use.


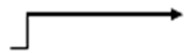


27. Have you ever engaged in **driven exercise** (i.e., exercise that feels excessive or compulsive)?

**YES NO**

**IF YES:**

28. At what age did you first engage in **driven exercise**? ______

29. Working back from today, how long has it been since you have engaged in **driven exercise**? (please answer in weeks)? If you have engaged in driven exercise this week, write zero ______

30. From the time that you first engaged in **driven exercise** until now, please choose the pattern that BEST describes your pattern of **driven exercise.**

0 = No driven exercise.


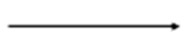


1 = Short episodes of driven exercise followed by longer intervals without driven exercise. Overall, driven exercise has been present 30% of the time or less.


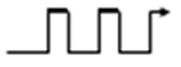


2 = Periods of driven exercise and periods without of approximate length. Overall, driven exercise has been present about 30-60% of the time.


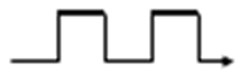


3 = Long periods of driven exercise with only short intervals without driven exercise. Overall, driven exercise has been present 60-90% of the time.


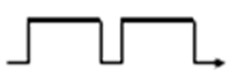


4 = Chronic driven exercise. Driven exercise has been present about 90-100% of the time. There have been no major intervals without driven exercise.


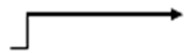


31. Have you ever felt very **concerned or preoccupied about your body weight or shape**?

**YES NO**

**IF YES:**

32. At what age do you think you first felt very **concerned or preoccupied about your body weight/shape**? ______

33. Working back from today, how long has it been since you have felt very **concerned or preoccupied about your body weight/shape**? (please answer in weeks)? If you currently feel this way, write zero ______

34. From the time that you first felt very **concerned or preoccupied about your body weight/shape** until now, please choose the pattern that BEST describes your pattern of **weight/shape concern or preoccupation.**

0 = No weight/shape concern or preoccupation.


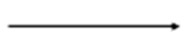


1 = Short episodes of weight/shape concern or preoccupation followed by longer intervals without weight/shape concern or preoccupation. Overall, weight/shape concern or preoccupation has been present 30% of the time or less.


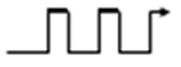


2 = Periods of weight/shape concern or preoccupation and periods without of approximate length. Overall, weight/shape concern or preoccupation has been present about 30-60% of the time.


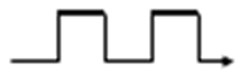


3 = Long periods of weight/shape concern or preoccupation with only short intervals without weight/shape concern or preoccupation. Overall, weight/shape concern or preoccupation has been present 60-90% of the time.


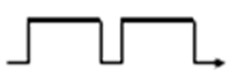


4 = Chronic weight/shape concern or preoccupation. Weight/shape concern or preoccupation has been present about 90-100% of the time. There have been no major intervals without weight/shape concern or preoccupation.


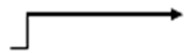


35. I consider myself to be RECOVERED.

**YES NO**

35. I consider myself to be IN RECOVERY.

**YES NO**

35. I consider myself to have an acute eating disorder (i.e., not yet be recovered or in recovery).

**YES NO**

**Supplemental Table 1.** Categorical responses from the Course of Illness scale by group.

| **Variables** | **Full Sample**  **(*n* = 75)** | **AN**  **(*n* = 31)** | **AN-WR**  **(*n* = 44)** |
| --- | --- | --- | --- |
| **General ED Pattern** |  |  |  |
| 0 | 2 (2.7%) | 1 (3.2%) | 1 (2.3%) |
| 1 | 4 (5.3%) | 1 (3.2%) | 3 (6.8%) |
| 2 | 17 (22.7%) | 3 (9.7%) | 14 (31.8%) |
| 3 | 24 (32.0%) | 11 (35.5%) | 13 (29.5%) |
| 4 | 28 (37.3%) | 15 (48.4%) | 13 (29.5%) |
| **Low Weight Pattern** |  |  |  |
| 0 | 6 (8.0%) | 3 (9.7%) | 3 (6.8%) |
| 1 | 20 (26.7%) | 3 (9.7%) | 17 (38.6%) |
| 2 | 14 (18.7%) | 5 (16.1%) | 9 (20.4%) |
| 3 | 24 (32.0%) | 14 (45.2%) | 10 (22.7%) |
| 4 | 11 (14.7%) | 6 (19.4%) | 5 (11.4%) |
| **Restrictive Eating Pattern** |  |  |  |
| 0 | 1 (1.3%) | 0 (0%) | 1 (2.3%) |
| 1 | 9 (12.0%) | 2 (6.5%) | 7 (15.9%) |
| 2 | 16 (21.3%) | 6 (19.4%) | 10 (22.7%) |
| 3 | 32 (42.7%) | 14 (45.2%) | 18 (40.9%) |
| 4 | 17 (22.7%) | 9 (29.0%) | 8 (18.2%) |
| **Weight/Shape Preocc. Pattern** |  |  |  |
| 0 | 2 (2.7%) | 1 (3.2%) | 1 (2.3%) |
| 1 | 8 (10.7%) | 1 (3.2%) | 7 (15.9%) |
| 2 | 7 (9.3%) | 3 (9.7%) | 4 (9.1%) |
| 3 | 21 (28%) | 6 (19.4%) | 15 (34.1%) |
| 4 | 37 (49.3%) | 20 (64.5%) | 17 (38.6%) |

Note: ED = eating disorder; Preocc = preoccupation. 0 = No endorsement of eating disorder/symptom; 1 = Short episodes of eating disorder/symptom followed by longer intervals without eating disorder/symptom; 2 = Periods of eating disorder/symptom and periods without of approximate length; 3 = Long periods of eating disorder/symptom with only short intervals without eating disorder/symptom; 4 = Chronic eating disorder symptom.

**Supplemental Table 2.** Group differences on clinical variables between AN and AN-WR groups.

| **Variables** | **AN**  Mean (SD) | **AN-WR**  Mean (SD) | **Mean Diff.**  (AN-AN-WR) | ***t*** | ***p*** |
| --- | --- | --- | --- | --- | --- |
| **BL BMI** | 17.24 (1.26) | 20.00 (1.00) | -2.76 | -10.16 | < .001* |
| **BL DRS** | 22.47 (7.76) | 12.50 (10.80) | 9.97 | 4.62 | < .001* |
| **BL Shape Concern** | 3.55 (1.34) | 3.00 (1.58) | 0.55 | 1.60 | .115 |
| **BL Weight Concern** | 3.26 (1.65) | 2.81 (1.62) | 0.45 | 1.16 | .252 |
| **ED Age** | 14.55 (5.02) | 15.64 (4.54) | -1.09 | -0.96 | .340 |
| **ED Duration** | 17.03 (14.76) | 7.59 (6.99) | 9.44 | 3.31 | .002* |
| **ED Course** | 3.23 (0.99) | 2.77 (1.03) | 0.45 | 1.92 | .059 |
| **LW Age** | 15.68 (6.52) | 17.68 (5.31) | -2.00 | -1.41 | .163 |
| **LW Duration** | 16.97 (16.56) | 5.05 (6.80) | 11.92 | 3.79 | < .001* |
| **LW Course** | 2.55 (1.21) | 1.93 (1.17) | 0.62 | 2.21 | .031* |
| **Restrict Age** | 15.90 (4.55) | 15.55 (3.67) | 0.36 | 0.36 | .718 |
| **Restrict Duration** | 17.94 (14.21) | 8.09 (6.99) | 9.84 | 3.57 | < .001* |
| **Restrict Course** | 2.97 (0.87) | 2.57 (1.04) | 0.40 | 1.80 | .077 |
| **Preocc Age** | 12.87 (6.54) | 13.32 (4.12) | -0.45 | -0.34 | .738 |
| **Preocc Duration** | 20.97 (14.06) | 9.89 (7.66) | 11.08 | 3.99 | < .001* |
| **Preocc Course** | 3.39 (1.02) | 2.91 (1.16) | 0.48 | 1.89 | .063 |

Note: BL = Baseline; BMI = Body Mass Index; ED = eating disorder; LW = low weight; Restrict = restrictive eating; Preocc = weight/shape preoccupation. *significant difference in means between AN and AN-WR (*p* < .05).

**Supplemental Table 3.** Estimated marginal means and pairwise comparisons for clinical variables between AN and AN-WR groups, adjusted for age.

| **Variable** | **Group** | **Adjusted Mean** (SE) | **Pairwise Diff.**  (AN – AN-WR) | ***t*** | ***p*** |
| --- | --- | --- | --- | --- | --- |
| **ED Duration** | AN | 12.4 (1.36) |  |  |  |
|  | AN-WR | 10.8 (1.12) | 1.6 | 0.87 | .390 |
| **ED Course** | AN | 3.25 (0.20) |  |  |  |
|  | AN-WR | 2.75 (0.16) | 0.50 | 1.90 | .062 |
| **LW Duration** | AN | 11.02 (0.98) |  |  |  |
|  | AN-WR | 9.24 (0.81) | 1.78 | 1.35 | .183 |
| **LW Course** | AN | 2.62 (0.23) |  |  |  |
|  | AN-WR | 1.88 (0.19) | 0.74 | 2.40 | .019* |
| **Restrict Duration** | AN | 12.5 (0.76) |  |  |  |
|  | AN-WR | 11.9 (0.63) | 0.54 | 0.52 | .603 |
| **Restrict Course** | AN | 2.97 (0.19) |  |  |  |
|  | AN-WR | 2.56 (0.16) | 0.41 | 1.61 | .112 |
| **Preocc Duration** | AN | 15.6 (0.94) |  |  |  |
|  | AN-WR | 13.6 (0.78) | 1.99 | 1.55 | .125 |
| **Preocc Course** | AN | 3.38 (0.21) |  |  |  |
|  | AN-WR | 2.91 (0.18) | 0.47 | 1.62 | .109 |

Note: ED = eating disorder; LW = low weight; Restrict = restrictive eating; Preocc = weight/shape preoccupation. *significant difference in estimated marginal means between AN and AN-WR after controlling for age using the emmeans r package (*p* < .05).

**Supplemental Table 4.** Exploratory inter-item correlations between onset, duration, and course of general eating disorder and specific symptoms.

| **Variables** | **ED Age** | **ED Course** | **ED Duration** | **LW Age** | **LW Course** | **LW Duration** | **Restrict Age** | **Restrict Course** | **Restrict Duration** | **Preocc Age** | **Preocc Course** |
| --- | --- | --- | --- | --- | --- | --- | --- | --- | --- | --- | --- |
| **ED Age** |  |  |  |  |  |  |  |  |  |  |  |
| **ED Course** | .38** |  |  |  |  |  |  |  |  |  |  |
| **ED Duration** | -.24* | .18 |  |  |  |  |  |  |  |  |  |
| **LW Age** | .45** | .25* | .02 |  |  |  |  |  |  |  |  |
| **LW Course** | .21 | .49** | .06 | .45** |  |  |  |  |  |  |  |
| **LW Duration** | -.39** | -.06 | .73** | -.20 | -.03 |  |  |  |  |  |  |
| **Restrict Age** | .53** | -.03 | -.28* | .23* | .09 | .06 |  |  |  |  |  |
| **Restrict Course** | .05 | .52** | .09 | .07 | .31** | .03 | .02 |  |  |  |  |
| **Restrict Duration** | -.35** | .06 | .91** | -.10 | -.02 | .92** | -.11 | .07 |  |  |  |
| **Preocc Age** | .17 | -.11 | -.14 | .03 | .01 | .17 | .54** | -.16 | .02 |  |  |
| **Preocc Course** | -.05 | .27* | .11 | .02 | .11 | .06 | .00 | .50** | .10 | .02 |  |
| **Preocc Duration** | -.22 | .12 | .86** | -.04 | -.01 | .85** | .00 | .16 | .93** | -.18 | .15 |

Note: ED = eating disorder; LW = low weight; Restrict = restrictive eating; Preocc = weight/shape preoccupation

**References**

Becker, A. E., Thomas, J. J., & Pike, K. M. (2009). Should non-fat-phobic anorexia nervosa be included in DSM-V?. *International Journal of Eating Disorders*, *42*(7), 620–635. <https://doi.org/10.1002/eat.20727>

Engel, S. G., Wonderlich, S. A., Crosby, R. D., Mitchell, J. E., Crow, S., Peterson, C. B., Le Grange, D., Simonich, H. K., Cao, L., Lavender, J. M., & Gordon, K. H. (2013). The role of affect in the maintenance of anorexia nervosa: evidence from a naturalistic assessment of momentary behaviors and emotion. *Journal of Abnormal Psychology*, *122*(3), 709–719. <https://doi.org/10.1037/a0034010>

Vanzhula, I., Hagan, K., Duck, S. A., Pan, I., Wang, E. Y., Steinglass, J., Attia, E., Wildes, J. E., Guarda, A. S., & Schreyer, C. (2024). Eating disorder symptom non-endorsers in hospitalised patients with anorexia nervosa: Who are they?. *European Eating Disorders Review*, *32*(4), 795–808. https://doi.org/10.1002/erv.3087
